# Genetic Correction of SOD1 Mutant iPSCs Reveals ERK and JNK Activated AP1 as a Driver of Neurodegeneration in Amyotrophic Lateral Sclerosis

**DOI:** 10.1016/j.stemcr.2017.02.019

**Published:** 2017-03-30

**Authors:** Akshay Bhinge, Seema C. Namboori, Xiaoyu Zhang, Antonius M.J. VanDongen, Lawrence W. Stanton

**Affiliations:** 1 Stem Cell and Regenerative Biology, Genome Institute of Singapore, Singapore 138672, Singapore; 2 Program for Neuroscience and Behavioral Disorders, Duke-NUS Medical School, Singapore 169857, Singapore; 3 NUS Graduate School for Integrative Sciences and Engineering, National University of Singapore, Singapore 119077, Singapore; 4 Department of Biological Sciences, National University of Singapore, Singapore 117543, Singapore

**Keywords:** ALS, SOD1, FUS, CRISPR-Cas9, p38, ERK, JNK, WNT, TP53, JUN

## Abstract

Although mutations in several genes with diverse functions have been known to cause amyotrophic lateral sclerosis (ALS), it is unknown to what extent causal mutations impinge on common pathways that drive motor neuron (MN)-specific neurodegeneration. In this study, we combined induced pluripotent stem cells-based disease modeling with genome engineering and deep RNA sequencing to identify pathways dysregulated by mutant SOD1 in human MNs. Gene expression profiling and pathway analysis followed by pharmacological screening identified activated ERK and JNK signaling as key drivers of neurodegeneration in mutant SOD1 MNs. The AP1 complex member JUN, an ERK/JNK downstream target, was observed to be highly expressed in MNs compared with non-MNs, providing a mechanistic insight into the specific degeneration of MNs. Importantly, investigations of mutant FUS MNs identified activated p38 and ERK, indicating that network perturbations induced by ALS-causing mutations converge partly on a few specific pathways that are drug responsive and provide immense therapeutic potential.

## Highlights

• Genome correction of SOD1 E100G mutation corrects ALS phenotypes in MNs
• Activation of MAPK, AP1, WNT, cell-cycle, and p53 signaling in ALS MNs
• Pharmacological screening uncovers ERK and JNK signaling as therapeutic targets
• Susceptibility of MNs to degeneration may be due to heightened JUN activity in MNs

## Teaser

In this article, Bhinge, Stanton, and colleagues use genome editing of patient-derived iPSCs to model ALS phenotypic defects in vitro. Transcriptomic analysis of disease MNs reveals activation of MAPK, AP1, WNT, cell-cycle, and p53 signaling in ALS MNs. Pharmacological screening uncovers activated ERK and JNK signaling as therapeutic targets in ALS.

## Introduction

Amyotrophic lateral sclerosis (ALS) is a devastating neurodegenerative condition characterized by a progressive loss of upper and lower MNs (Therrien et al., 2016). There is no cure to halt or reverse the degeneration and the median patient survival is 3–5 years (Robberecht and Philips, 2013). Approximately 20% of ALS cases are familial with mutations identified in greater than 20 genes across diverse cellular functions (Andersen and Al-Chalabi, 2011, Sreedharan and Brown, 2013). Several mutations in the superoxide dismutase 1 (*SOD1*) gene have been linked to ALS (Al-Chalabi et al., 2012). Although rodent models of ALS have provided valuable insights into disease pathophysiology, the relevance of these findings to human ALS has been questioned due to the inherent species-specific differences as well as the fact that most rodent models overexpress the mutant proteins at non-physiological levels (Gladman et al., 2012). Spinal motor neurons (MNs) differentiated from patient-specific induced pluripotent stem cells (iPSCs) have provided researchers with access to human MNs bearing ALS-associated mutations in the context of the patient's genetic background, thereby providing a powerful in vitro human model of disease that can complement existing transgenic rodent models (Faravelli et al., 2014). In this study, we used patient-derived iPSCs bearing the SOD1 E100G mutation combined with genome engineering, neuronal differentiation, and genomics to identify pathways dysregulated in ALS. More importantly, we demonstrate that modulation of these pathways by pharmacological intervention results in a reduction of disease-phenotypic severity.

## Results

**Figure 1:**
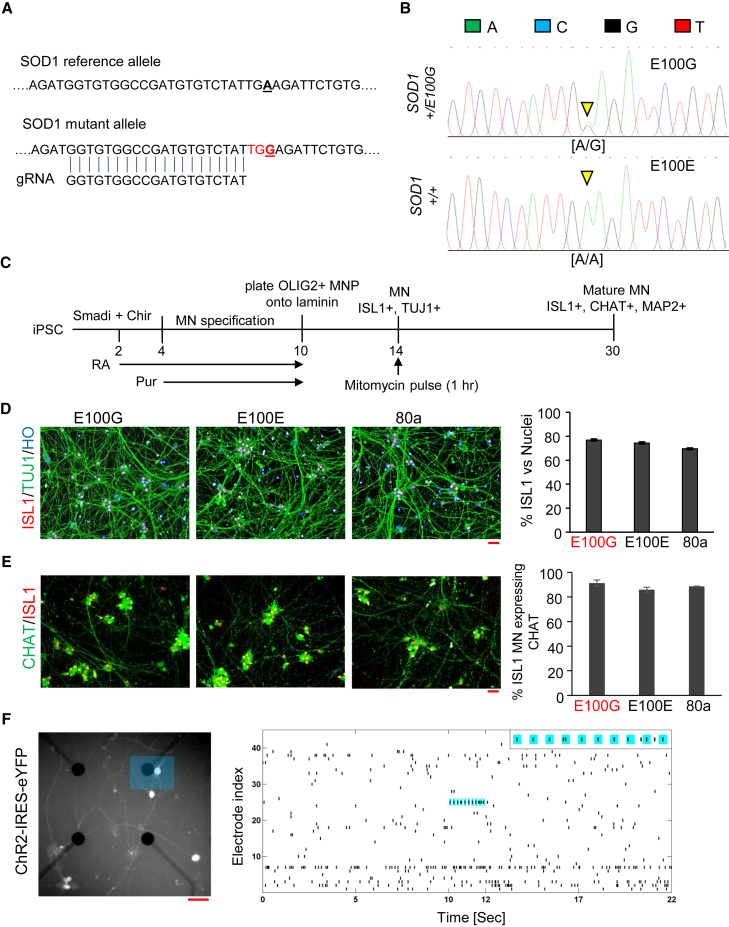
Generation of Isogenic Controls and Spinal Motor Neurons (A) Guide-RNA design and targeting of the SOD1 mutant allele. Genomic sequence around the SOD1 mutant allele is shown with the reference and mutant allele (bold and underlined). The guide RNA is shown aligned with the mutant locus with the PAM recognition sequence highlighted in red. (B) Chromatogram showing CRISPR-Cas9-mediated genome correction of *SOD1*+/E100G (designated E100G) to *SOD1*+/+ (designated E100E). The inverted yellow triangles indicate the heterozygous point mutation A/G (upper panel) and the corresponding homozygous A/A genotype (lower panel) upon genome correction. (C) Schematic illustrating differentiation of iPSCs into spinal MNs. Smadi, dual SMAD inhibition using SB431542 + LDN193189; Chir, CHIR99021; RA, retinoic acid; Pur, purmorphamine. (D) Differentiation of MNs from iPSCs. Neurons were immunostained for ISL1 (red) and TUJ1 (green). Nuclei were stained with Hoechst 33452 (blue). E100G, *SOD1*+/E100G; E100E, *SOD1*+/+; 80a, iPSCs from a healthy individual used as a control. (E) Quantitation of MNs staining positive for CHAT. Neurons were immunostained for ISL1 (red) and CHAT (green). (n = 3, error bars indicate SEM). (F) Left: Representative fluorescent image of the MEA to visualize eYFP expression. MNs co-expressed Channel Rhodopsin-2 (ChR2) with eYFP. The blue rectangle highlights the area stimulated with a 473 nm laser at 5 Hz (50% duty cycle) for 2 s. Right: Raster plots of action potentials recorded extracellularly from active electrodes of the MEA. In total, 42 active channels containing at least two spikes were plotted. The electrode adjacent to the optically stimulated neuron (blue rectangle in the left panel) detected spiking activity synchronized to the 5 Hz stimulus protocol. Inset: enlarged plot showing light-evoked spikes with blue background indicating when the light was on (see also Figure S1). All scale bars indicate 50 μM.

Disease modeling using iPSCs can be confounded by phenotypic variation resulting from genetic differences between individual iPSC lines (Merkle and Eggan, 2013). To overcome this obstacle, we sought to generate isogenic cell lines by targeted correction of the point mutation in the *SOD1* iPSC using the CRISPR-Cas9 system (Hendriks et al., 2016). We designed a guide RNA to specifically target the mutant allele, taking advantage of the observation that the A > G mutation in the *SOD1* locus creates a PAM recognition sequence not present in the reference allele (Figure 1A). In addition, the guide RNA was chosen such that Cas9 would create a double-stranded break within 5–6 bp of the targeted mutation, thereby increasing the efficiency of incorporating the reference allele provided via a donor DNA oligonucleotide (Figure 1A and Experimental Procedures). Correction of the heterozygous point mutation in the *SOD1* gene was confirmed by PCR amplification of the targeted genomic regions followed by capillary sequencing (Figure 1B). In addition, the corrected iPSCs displayed a normal karyotype (Figure S1A).

We differentiated the diseased and corrected iPSCs as well as an iPSC line derived from a healthy individual (designated as 80a) into spinal motor neurons by adapting a recently published protocol capable of generating MNs with high efficiency (Figure 1C; Maury et al., 2015). For all the iPSC lines used in our study, we were able to generate OLIG2+ motor neuron progenitors at day 10 and post-mitotic ISL1+/TUJ1+ MNs at day 14 (Figures S1B and S1C). At day 30 of differentiation, all iPSC lines generated ISL1+/TUJ1+ spinal MNs with efficiencies >70%, similar to that observed previously (Figure 1D). In addition, greater than 80% of the ISL1+ MNs at day 30 also expressed CHAT, a marker of mature MNs, and both ISL1+ MNs as well as ISL1− non-MNs expressed MAP2, a pan-neuronal marker expressed in mature neurons (Figures 1E and S1D; Sances et al., 2016). Further, our iPSC-derived MNs were electrophysiologically active and responded to optogenetic stimulation (Figure 1F). Lastly, when co-cultured with rat cortical neurons (Figure S1E), MNs displayed synchronized neural activity as measured by the genetically encoded Ca+ indicator GCaMP6 (Figure S1F). Importantly, the observed MN neural activity was synchronous with the cortical neuronal activity (Figure S1G), indicating that our MNs were capable of accepting synaptic input from the cortical neurons. Taken together, the marker expression and neural activity indicated that our iPSC-derived MNs were mature and functionally active.

**Figure 2:**
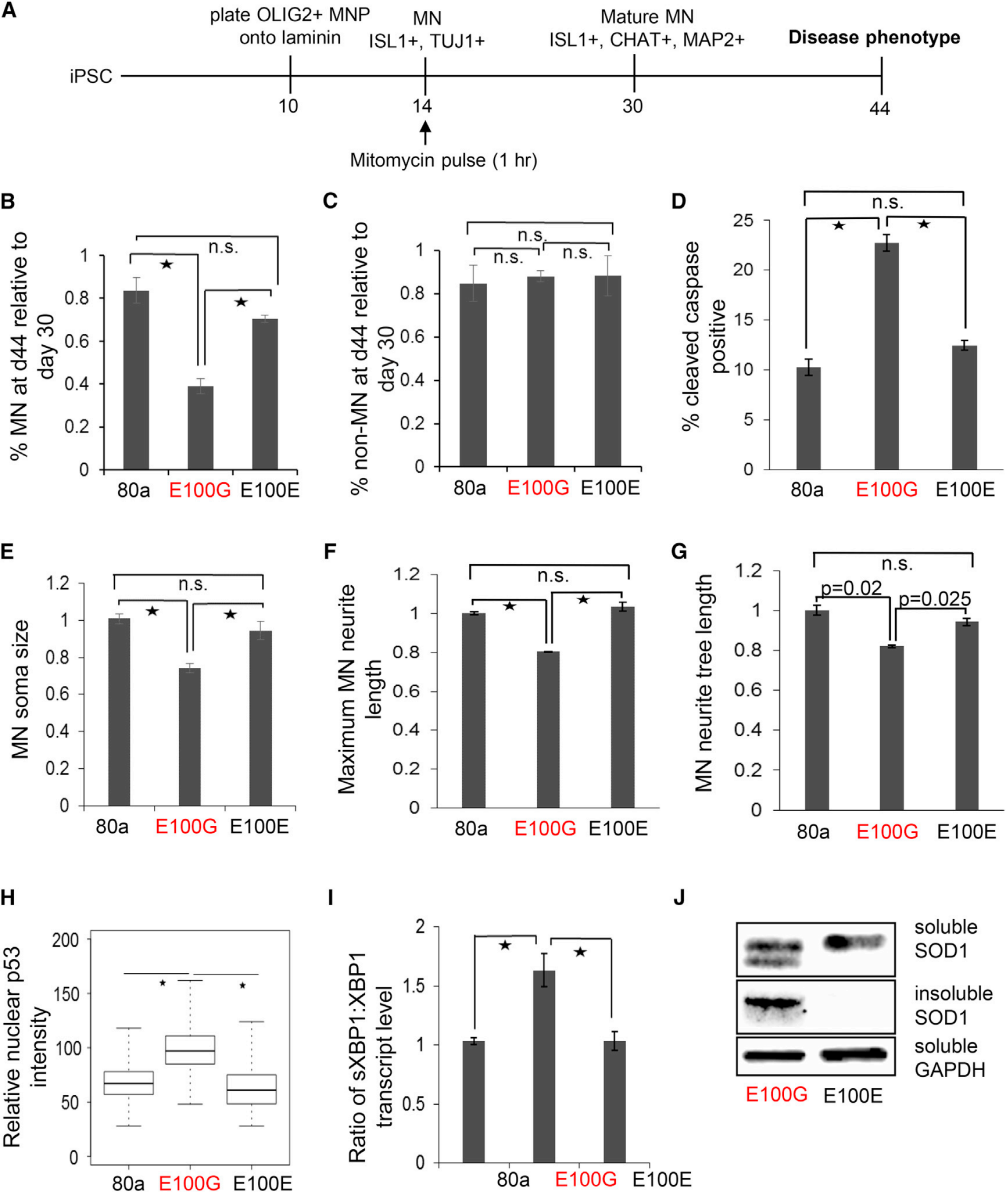
Modeling ALS In Vitro Using iPSC-Derived MNs (A) Schematic depicting the disease modeling protocol. (B) ALS MNs display a significant loss in survival compared with control MNs when plated at low density. Correction of the ALS-associated mutation rescues the observed phenotype. MNs were identified as ISL1+/TUJ1+ neurons. (C) ALS or control non-MNs, identified as ISL1-/TUJ1+, do not display a survival deficit. (D) ALS MN cultures at day 37 display a higher percentage of cells undergoing apoptosis as measured by cleaved caspase activity. (E) Quantitation of soma size for day 44 MNs. Soma size was normalized to control day 44 MNs. (F) Quantitation of maximum neurite length for day 44 MNs. Data were normalized to control day 44 MNs. (G) Quantitation of neurite tree length for day 44 MNs. Data were normalized to control day 44 MNs. (H) Immunofluorescence intensities of nuclear p53 in day 30 MNs. Values were normalized to control MNs. Data from three independent replicates were pooled to generate the boxplot and estimate the p values. (I) qRT-PCR data to measure the *sXBP1:XBP1* ratio, an indicator of ER stress in day 37 MNs. Ratios were normalized to data from control MNs. (J) Western blot assay showing levels of detergent-soluble and insoluble SOD1 in ALS and control MN lysates at day 30. (B–I) n = 3, error bars indicate SEM; ^∗^p < 0.01; n.s., not significant; p values were estimated using two-tailed Student’s t test. See also Figure S2.

Since the over-arching phenotype observed in ALS patients is the specific loss of MNs, we asked whether ALS MNs would reveal disease-associated decline of survival in vitro. ALS is an adult-onset disease, therefore we monitored survival of MNs after they had attained maturity. Accordingly, we followed MN survival in low-density cultures from day 30 to day 44 (Figure 2A). ALS mutant MNs showed a significant decline in survival compared with the healthy control MNs (Figure 2B). On the other hand, genetic correction of the disease MNs significantly improved survival to a level comparable with wild-type MNs, indicating that correction of the mutation had ameliorated the disease phenotype (Figure 2B). Importantly, this decline in survival was not observed in ISL1−/TUJ1+ non-MN (Figure 2C), thereby recapitulating the MN-specific loss observed in ALS. In addition, we observed increased cleaved caspase activity in disease MN cultures (day 37) compared with healthy or isogenic control cultures, indicating that the loss of diseased MNs was, in part, due to apoptosis (Figure 2D).

We observed that increased apoptosis in diseased MNs was also accompanied by morphological changes that were consistent with observations of postmortem spinal tissue from ALS patients (Kiernan and Hudson, 1991). Morphometric analysis of our in vitro ALS model revealed a reduction in the soma size, maximum neurite length, as well as average neurite tree length in mutant MNs at day 44 compared with the control MNs, while genetic correction of the mutation improved these morphological characteristics in the isogenic MNs (Figures 2E–2G). We also observed significantly higher levels of the tumor suppressor p53 (TP53) in the nuclei of mutant SOD1 MNs compared with both the healthy control and isogenic MNs (Figure 2H), which is concordant with activated p53 observed in ALS postmortem spinal tissue as well as rodent models of ALS (Qiu et al., 2014, Ranganathan and Bowser, 2010). Studies on the SOD1 G93A mouse model of ALS, as well as a recent iPSC model of SOD1 ALS, have revealed heightened endoplasmic reticulum (ER) stress in ALS MNs (Kiskinis et al., 2014, Nishitoh et al., 2008). In cells undergoing ER stress, IRE1 splices XBP1 to generate the active spliced form of the transcription factor (sXBP1), while PERK activates the transcription factor ATF4, which leads to upregulation of ATF3 and CHOP (Jiang et al., 2004, Xiang et al., 2016). To assay whether our in vitro model revealed an increase in ALS-related ER stress, we measured the ratio of spliced *XBP1* to total *XBP1* transcript levels (*sXBP1:XBP1*) via qRT-PCR. We found an increased *sXBP1:XBP1* ratio in SOD1 MNs compared with the control MNs that normalized upon genetic correction (Figure 2I). In addition, we observed significantly higher levels of ATF3 and CHOP in SOD1 MN cultures compared with both the control and isogenic MNs (Figures S2A and S2B), indicating that the ALS iPSC-derived MNs displayed an increased ER stress response.

The observed correction of the phenotypes was not due to altered expression of *SOD1* transcripts between the diseased and isogenic MNs (Figure S2C). Aggregation of mutant SOD1 into insoluble inclusions is commonly observed in SOD1 ALS (Taylor et al., 2016). Treatment of MN cultures with the proteasome inhibitor MG132 revealed significant levels of insoluble SOD1 in the diseased MNs, although similar levels of soluble SOD1 protein were observed between the diseased and isogenic controls (Figure 2J). Interestingly, an extra band that migrated slightly faster was detected for SOD1 specifically in the mutant MN soluble fraction, probably representing misfolded protein (Chen et al., 2014). Taken together, the phenotypes observed in our in vitro model closely recapitulate findings from ALS postmortem tissue and rodent models. In addition, amelioration of the phenotypes upon genetic correction indicated that the observed phenotypes resulted from the underlying mutation and were not due to genotypic variations between the iPSC lines.

**Figure 3:**
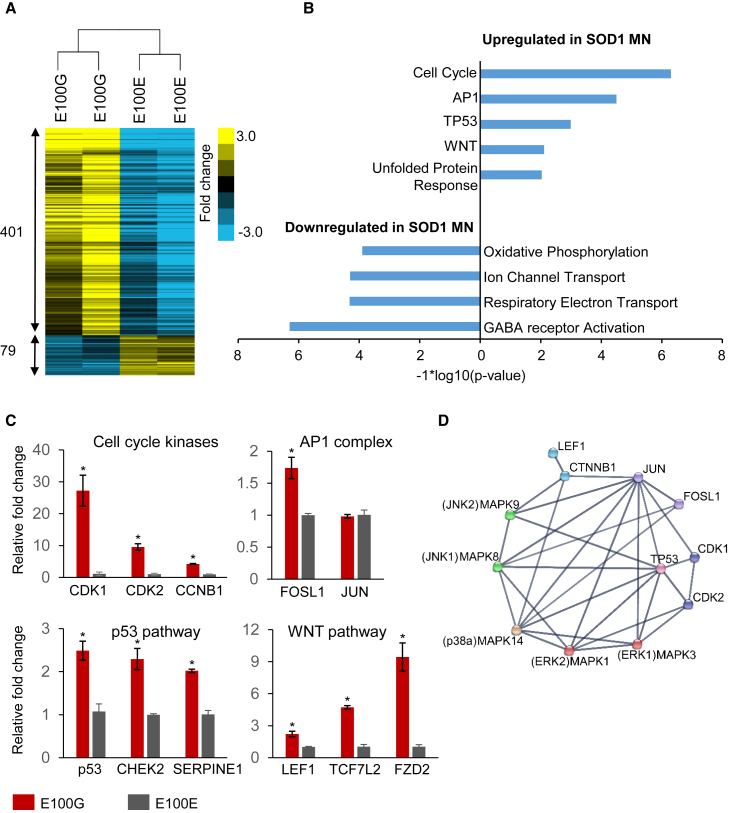
RNA-Seq Analysis Identifies Signaling Pathways Dysregulated in ALS MNs (A) Dendrogram showing clustering of diseased and isogenic corrected samples based on transcriptional changes detected by RNA-seq. The number of genes differentially regulated in ALS MNs compared with isogenic control MNs are displayed. (B) GSEA detected gene sets activated and repressed in SOD1 MNs. (C) qRT-PCR to confirm activation of genes identified via RNA-seq. n = 3, error bars indicate SEM; ^∗^p < 0.01; p values were estimated using two-tailed Student’s t test. (D) Network analysis of gene sets activated in SOD1 MNs. Data were obtained from the STRING database (Szklarczyk et al., 2015).

To understand the molecular pathways driving MN loss, we performed genome-wide transcriptome profiling of the diseased and corrected MNs using RNA-seq. We chose to use young MNs (day 30) to avoid transcriptional changes associated with cell death. Unsupervised hierarchical clustering of the RNA-seq data showed that the diseased MNs segregated distinctly from the corrected ones, indicating that correction of the SOD1 mutant allele had induced significant changes in the transcriptome (Figure 3A). We identified 480 genes in the SOD1 dataset that were differentially regulated (p < 0.01) with a majority of the genes being activated in SOD1 mutant MNs (Figure 3A). To enable sensitive detection of altered regulatory pathways from the differential gene expression data, we performed gene set enrichment analysis (GSEA), which relies on ranking genes in order of significance and avoids setting hard thresholds to identify differentially expressed genes (Mootha et al., 2003, Subramanian et al., 2005).

GSEA identified several pathways as differentially regulated in ALS MNs. For SOD1, significantly upregulated pathways were associated with p53 activation, cell-cycle regulation, WNT signaling, AP1 activation, and the unfolded protein response (UPR) (Figure 3B). Strikingly, the downregulated gene sets were associated with mitochondrial function, including electron transport, ATP synthesis, and oxidative phosphorylation (Figure 3B). Mitochondrial defects and an activated UPR have previously been shown to be associated with the SOD1 A4V mutation in MNs (Kiskinis et al., 2014). Here, we find that the SOD1 E100G mutation results in similar molecular defects in MNs. In addition, genes involved in ion channel transport, especially γ-aminobutyric acid receptors, were also downregulated, which could partially explain the previously reported excitotoxicity in SOD1 MNs (Wainger et al., 2014) (Figure 3B).

For further investigation, we decided to focus on the pathways that were activated in ALS MNs. qRT-PCR confirmed differential expression of several genes identified from RNA-seq analysis and representative of pathways found to be activated by GSEA (Figure 3C). We hypothesized that the activation of seemingly disparate pathways in mutant SOD1 MNs could result from the differential activation of a few upstream effectors. When placed in the context of a functional gene network, we observed that several of these pathways shared functional interactions with MAPK signaling and displayed extensive cross-talk between the identified pathways (Figure 3D). For example, the AP1 complex protein JUN can be directly activated by the ERK (MAPK1,3), JNK (MAPK8,9), and p38 (MAPK14) kinases, while the WNT mediator CTNNB1 (β-catenin) can be activated by the p38 (MAPK14) kinase. In addition, JUN as well as the cell-cycle mediators CDK1 and CDK2 have functional interactions with TP53 (Figure 3D). The network analysis raised the possibility that activation of a select few pathways may be sufficient to initiate the cascade of signaling perturbations observed from our RNA-seq data.

**Figure 4:**
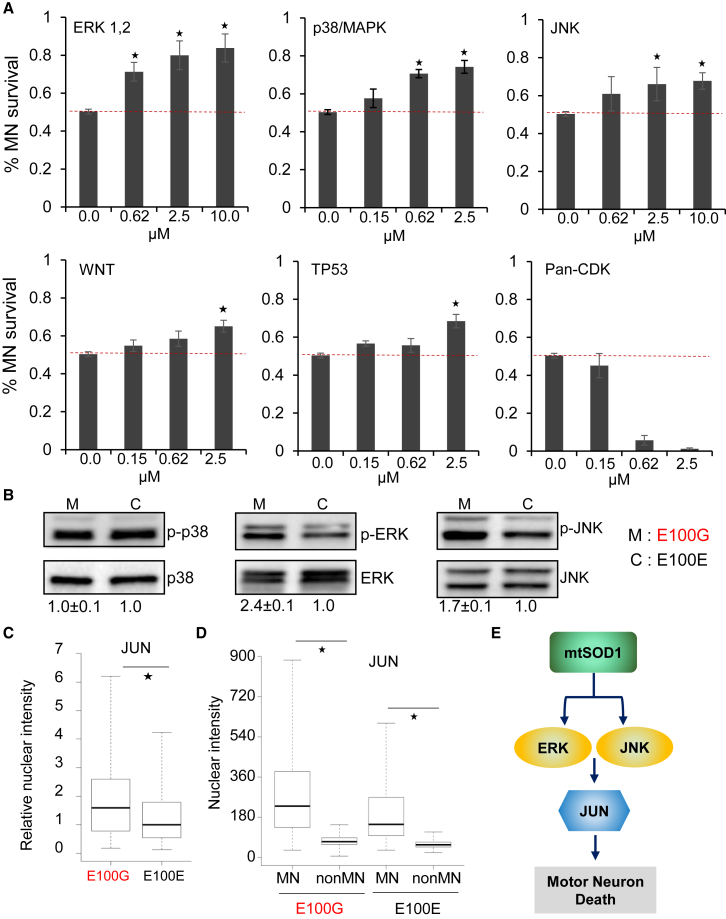
Identification of Pathways Driving Neurodegeneration in ALS MNs (A) Quantitation of ISL1-positive mutant SOD1 MNs after treatment with small-molecule inhibitors of the indicated pathways. n = 3, error bars indicate SEM; ^∗^p < 0.01; p values were estimated using two-tailed Student’s t test. (B) Western blot analysis showing increased levels of phosphorylated ERK and JNK in mutant SOD1 MNs. Percentages relative to respective isogenic controls are displayed below each blot. Values underlined indicate increased levels of the detected proteins. Lysates from two independent replicates were pooled and assayed in triplicate. Data indicate means ± SEM. (C) Boxplot displaying nuclear intensities of p-JUN in ALS mutant MNs normalized to the respective isogenic controls. Data from three independent replicates were pooled to generate the boxplot and estimate the p values. ^∗^p < 0.01; p values were estimated using two-tailed Student’s t test. (D) Boxplot displaying nuclear intensities of p-JUN in mutant SOD1 MNs and non-MNs as well as isogenic control MNs and non-MNs. Data from three independent replicates were pooled to generate the boxplot and estimate the p values. ^∗^p < 0.01; p values were estimated using two-tailed Student’s t test. (E) Schematic depicting pathways activated by mutant SOD1 that drive neurodegeneration. See also Figure S3.

To investigate whether the activated pathways identified in ALS MNs were causal or merely associated with the disease phenotype, we assayed MN survival in vitro after pharmacological inhibition of the identified pathways. MNs differentiated from mutant SOD1 iPSCs were treated with small-molecule inhibitors targeting the p38, ERK, JNK, and CDK kinases, as well as p53 and WNT pathways, at the indicated concentrations starting at day 30 and continued until day 44 (Figure 4A, Table S1). Untreated (vehicle only control) SOD1 MNs showed a significant loss of survival with ∼50% of the MNs lost at day 44 compared with day 30 (Figure 4A). Strikingly, inhibition of the AP1 complex, especially via targeting ERK signaling, reduced MN loss in a dose-dependent manner such that only 16% of the MNs were lost at day 44 at the highest concentration used (Figure 4A). Inhibition of p38/MAPK signaling similarly restricted mutant SOD1 MN loss to 25% (Figure 4A). Treatment of SOD1 MNs with a JNK inhibitor or an inhibitor of the WNT pathway had a significant but modest effect on SOD1 MN survival compared with the control treatment (Figure 4A). Surprisingly, inhibition of the p53 pathway displayed only a modest improvement on the survival of ALS MNs, suggesting that p53 activation may play a minor role in driving neurodegeneration in ALS (Figure 4A). The CDK inhibitor did not promote survival of ALS MNs and, unexpectedly, was toxic to MNs at the higher concentrations used (Figure 4A). In concordance with our phenotypic screen, we observed activation of ERK1/2 and JNK1/2 in the SOD1 mutant MNs (Figure 4B). Surprisingly, contrary to our expectation, we did not observe activation of p38 in mutant SOD1 MNs compared with isogenic control MNs (Figure 4B).

We performed immunofluorescence assays in ALS and isogenic control MNs to confirm activation of the AP1 and WNT pathways. For a given target protein (i.e., JUN), we measured the median fluorescent signal in the nuclei of diseased and isogenic control MNs and plotted the distribution of intensities (Figure 4C). Immunofluorescence assays confirmed increased nuclear localization of the AP1 complex member JUN as well as β-catenin in mutant SOD1 MNs compared with the isogenic control MNs (Figures 4C and S3A), confirming activation of the AP1 and WNT pathways in ALS MNs.

ALS is characterized by selective loss of MNs, while other neurons remain viable even though they harbor the same germline mutations. Using our in vitro model, we asked if there was differential activation of signaling pathways in MNs and non-MNs that might confer disease-specific loss of MNs. We analyzed nuclear localization of JUN and β-catenin in MNs (ISL1+) and non-MNs (ISL1−). Strikingly, we found that the levels of nuclear JUN were almost five times higher in MNs compared with non-MNs (Figure 4D). This difference between MNs and non-MNs was evident even in the isogenic controls and was heightened in the SOD1 cultures (Figure 4D). This observation suggests a mechanism where MNs require an active but tightly regulated AP1 pathway for normal homeostasis that is not required by non-MNs. Mutant SOD1 might cause hyperactivation of the AP1 pathway via ERK and JNK activation, perturbing the homeostasis and thereby driving neurodegeneration (Figure 4E). Although we observed a modest increase in nuclear JUN in the non-MN population, the absolute levels of the protein might not be sufficient to perturb homeostasis. This might explain the selective loss of MNs due to over-activation of the AP1 pathway. We observed a similar trend for WNT activation, where MNs displayed higher WNT activity compared with non-MNs, although the difference was less striking compared with that observed for JUN (Figure S3B).

**Figure 5:**
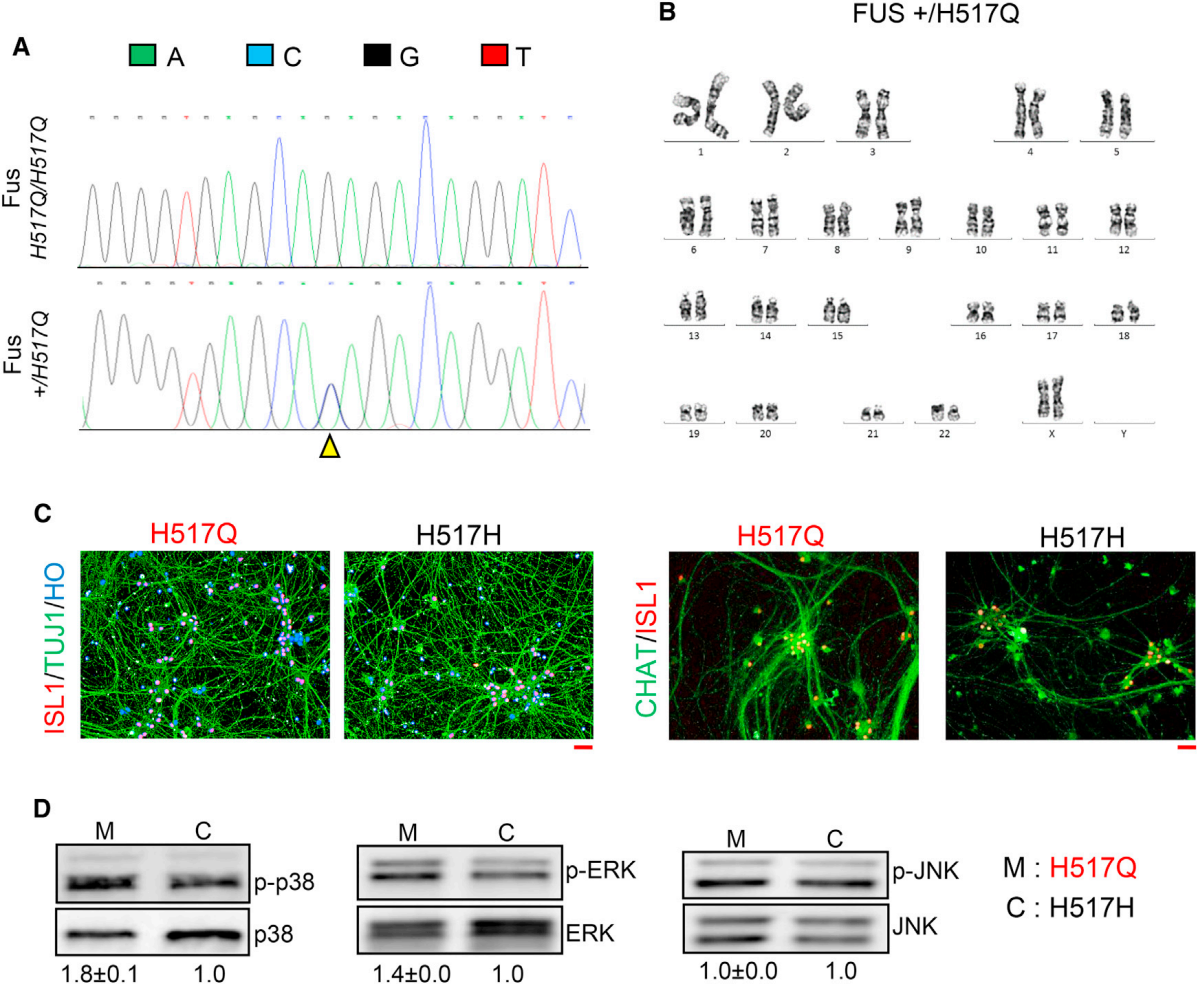
Mutant FUS MN Display Activated MAPK Signaling (A) Chromatogram showing correction of homozygous FUS *H517Q/H517Q* iPSCs (designated H517Q) to heterozygous FUS*+/H517Q* (designated H517H). The yellow triangle indicates the position of the homozygous point mutation G/G (upper panel) and the corresponding heterozygous C/G genotype (lower panel) upon genome correction. (B) Isogenic corrected iPSCs display a normal karyotype. (C) Mutant and isogenic corrected iPSCs differentiate into ISL1+/TUJ1+ MNs as well as ISL1+/CHAT+ MNs with similar efficiencies. (D) Western blot assay displaying activated p38 and ERK in mutant FUS MNs compared with the isogenic controls. JNK was not found to be activated in mutant FUS MNs. Lysates from two independent replicates were pooled and assayed in triplicate. Data indicate means ± SEM. All scale bars indicate 50 μM.

Next, we asked whether pathways driving neurodegeneration in SOD1 MNs were also activated by other ALS-associated mutations. To answer this question, we sought to analyze the MAPK pathway in MNs bearing mutations in the FUS gene. We obtained iPSCs from a patient homozygous for the recessive H517Q mutation in the *FUS* gene (Kwiatkowski et al., 2009) and corrected this recessive mutation to heterozygous wild-type using the CRISPR-Cas9 system. Correction of the homozygous point mutation was confirmed by PCR amplification of the targeted genomic region followed by DNA sequencing (Figure 5A). We further confirmed that the corrected FUS iPSCs displayed a normal karyotype (Figure 5B). We were able to differentiate both the mutant and corrected FUS iPSCs into ISL1+/TUJ1+ spinal MNs with efficiencies similar to those obtained with the SOD1 iPSCs (H517Q/H517Q, 73% ± 1.7%; +/H517Q, 70% ± 0.74%; mean ± SEM) (Figure 5C). In addition, greater than 85% of ISL1+ MNs (day 30) expressed the mature MN marker CHAT (Figure 5C), similar to efficiencies obtained using SOD1 and control iPSCs. To investigate activation of the MAPK pathway, we assayed expression of individual MAPK family members in protein lysates derived from the mutant and corrected FUS MNs. Strikingly, we found activation of the p38 kinase and ERK kinase in the FUS mutant MNs, while the JNK kinase was not activated (Figure 5D). These observations indicate that MAPK activation is a common feature related to both SOD1 and FUS mutations, although these mutations activate distinct members of the MAPK family.

## Discussion

Although animal models have provided valuable mechanistic insights into the pathophysiology of ALS, there has been a lack of success in translating these findings to therapies (Gladman et al., 2012). This is perhaps due to species-specific differences between humans and rodents, or because there are issues with the animal model itself where a phenotype is observed only when the mutant protein is overexpressed at non-physiological levels. The use of patient-derived iPSCs promises to circumvent some of these obstacles by providing human neurons expressing the mutant protein at physiological levels in the relevant genetic background. However, one of the caveats of iPSC-based disease modeling is the phenotypic variation observed due to the underlying genetic differences between different iPSC lines. In addition, since iPSC-derived neurons are considered to be fetal-like (Sances et al., 2016), it is important to establish that the in vitro model is capable of recapitulating disease phenotypes observed in ALS patients.

To exclude the possibility of observing phenotypic differences due to genetic variation in the iPSC lines, we generated isogenic iPSC lines by correcting the point mutation in the *SOD1* genomic locus using CRISPR-Cas9 genome editing technology. Next, we confirmed the maturity of our iPSC-derived neuronal cultures by assaying for expression of CHAT and MAP2, bona fide markers of MN maturity. Further, by assaying for phenotypes that were observed in either ALS postmortem tissue or rodent models, we established that our in vitro model faithfully recapitulates specific aspects of the disease.

Having established that we were able to capture ALS disease pathophysiology in our in vitro system, we sought to uncover additional pathways dysregulated in ALS MN by deep RNA-seq. Analysis of the RNA-seq data identified several pathways commonly dysregulated in ALS MNs. For instance, activation of cell-cycle genes and p53 was observed in mutant SOD1 MNs. Since neurons are post-mitotic, reactivation of the cell cycle in neurons results in activation of the apoptotic pathway, partly in a p53-dependent manner (Herrup and Yang, 2007). Hence, we expected that inhibition of either p53 or the cell-cycle program would result in increased survivability of ALS MNs. However, inhibition of the cell-cycle pathway by targeting the CDK proteins diminished cell survival. This suggests that CDKs may have cell-cycle-independent functions in MN essential for homeostasis. Inhibition of p53 resulted in a modest improvement in survival, indicating that p53 may not be a driver of neurodegeneration, an observation supported by rodent ALS models (Kuntz et al., 2000).

On the other hand, pharmacological inhibition of MAPK signaling significantly improved survival for mutant SOD1 MN. In addition, we observed activation of ERK and JNK kinases in addition to JUN, an AP1 complex member and a direct target of both ERK and JNK signaling. It must be noted that mutant SOD1 MNs responded favorably to ERK inhibition but only modestly to JNK inhibition. Even though p38 kinase was not observed to be activated in SOD1 MNs compared with control, inhibition of p38/MAPK significantly improved mutant SOD1 MN survival almost on a par with ERK inhibition, possibly by lowering JUN levels. Overall, our results indicate that mutant SOD1 activates the AP1 pathway via ERK and JNK signaling to drive neurodegeneration. It must also be noted that no single inhibitor was able to completely rescue survival of ALS MNs, suggesting that a combination of inhibitors targeting different MAPK proteins might be the best strategy to halt neurodegeneration.

How does mutant SOD1 activate MAPK signaling? Recently, it was shown that ER stress leads to activation of JNK signaling via phosphorylation of HIPK2 in the SOD1 G93A mouse model of ALS. Activation of HIPK2 and JNK closely correlated with SOD1 aggregation and cell death, suggesting a mechanism whereby increased SOD1 aggregation leads to a heightened ER stress that in turn causes cell death via activation of JNK (Lee et al., 2016). It is possible that the ER stress may result in activation of other members of the MAPK family, including ERK, via hitherto unknown intermediary kinases. This would make the ER stress pathway an attractive target to identify ALS therapeutics. However, it must be noted that inhibiting the ER stress pathway via RNAi or pharmacological inhibition in an iPSC model of SOD1 ALS led to only modest improvement in MN survival (Kiskinis et al., 2014). On the other hand, FUS-R521C transgenic mice did not display any increase in ER stress markers or JNK activation in spite of showing progressive neurodegeneration (Lee et al., 2016). Consistent with these observations, we find that MNs derived from mutant FUS iPSCs do not display activation of JNK signaling. However, these mutant FUS MNs show activation of other members of the MAPK family, namely p38 and ERK. Taken together, this suggests that the ER stress may contribute toward activating the MAPK pathway in ALS, although to a minor extent.

Muscle fasciculations are commonly observed in ALS patients and are related to underlying axonal hyperexcitability (Kanai et al., 2006). MN hyperexcitability has also been observed in vitro in a SOD1 A4V iPSC model, and inhibiting neuronal excitability via retigabine enhanced MN survival (Wainger et al., 2014). On the other hand, MNs derived from iPSCs bearing the hexanucleotide repeat expansions in *C9ORF72* showed diminished activity (Devlin et al., 2015, Sareen et al., 2013). A recent study elegantly reconciled these two contradictory findings, suggesting that ALS MNs display hyperexcitability in the early stages but become hypoexcitable as the disease progresses (Devlin et al., 2015). Interestingly, neuronal firing has been shown to activate the ERK pathway, while a hyper-active ERK pathway has been shown to result in epilepsy (Guo et al., 2014, Nateri et al., 2007). This suggests a positive feedback between neuronal firing and activation of the ERK signaling cascade. Hyperexcitability in early-stage ALS MNs could result in activation of ERK signaling, thereby triggering the auto-feedback loop leading to neurodegeneration. Accordingly, inhibiting this loop either by targeting the ERK pathway or neuronal excitability promotes MN survival. It would be interesting to see whether inhibiting the ERK pathway or other members of the MAPK family would result in decreased neuronal excitability or whether inhibiting neuronal firing via retigabine reduces MAPK activity.

It must be noted that protein aggregation and an elevated ER stress have also been identified in other familial forms of ALS, including the most common mutation observed in *C9ORF72* (Taylor et al., 2016, Dafinca et al., 2016, Kaus and Sareen, 2015). In addition, many sporadic ALS cases commonly display TDP-43 protein inclusions in spinal MNs (Mackenzie et al., 2007), and these have been shown to correlate with ER stress and JNK activation (Lee et al., 2016). Further, neuronal hyperexcitability has been established as a recurring theme across sporadic as well as familial forms of ALS (Geevasinga et al., 2016). Hence, it is reasonable to suppose that a combination of the ER stress mediated by TDP-43 inclusions and neuronal hyperexcitability might contribute toward activation of the MAPK pathway in other forms of ALS as well.

Due to differences in iPSC generation and MN differentiation protocols, the range and extent of phenotypes observed in ALS iPSC models are expected to vary. However, comparison across our iPSC model and other published ALS iPSC models revealed shared features among MNs bearing different ALS mutations. MNs bearing mutant SOD1 and TDP43 display decreased survival and increased apoptosis in extended cultures (Bilican et al., 2012, Chen et al., 2014, Kiskinis et al., 2014; Figures 2B and 2D). MNs differentiated from *C9ORF72* mutant iPSCs did not display survival deficits in vitro under standard culture conditions (Devlin et al., 2015) but displayed enhanced susceptibility to cellular stressors (Donnelly et al., 2013, Haeusler et al., 2014). Interestingly, a recent report using patient-derived iPSCs bearing C9ORF72 expansion of greater than 500 repeats displayed increased neuronal death and apoptosis in vitro (Dafinca et al., 2016). Apoptotic MNs also display defects in soma size and neurite length (Chen et al., 2014, Kiskinis et al., 2014). Although neurite defects have not been observed in *C9ORF72* mutant MNs, interference with actin dynamics via interaction of C9ORF72 with cofilin can be expected to result in axonal defects (Sivadasan et al., 2016). An activated p53 response was observed in *C9ORF72* mutant MNs (Lopez-Gonzalez et al., 2016) similar to our observation of an increased nuclear p53 in SOD1 MNs compared with the control and isogenic corrected MNs. ER stress has also been detected in MNs derived from mutant SOD1 and C9ORF72 iPSCs in parallel to the detection of SOD1 aggregation and RAN dipeptide accumulation, respectively (Dafinca et al., 2016, Kiskinis et al., 2014). On the other hand, defects in nucleocytoplasmic transport that have been observed in C9ORF72 ALS have yet to be detected in other familial forms of ALS (Freibaum et al., 2015, Zhang et al., 2015).

An important yet unanswered question in ALS disease pathophysiology is the targeted loss of MNs but relative sparing of non-MNs. Strikingly, we found that the AP1 complex member JUN was expressed at significantly higher levels in MNs compared with non-MNs, suggesting a role of JUN in maintaining MN homeostasis. This observation is in concordance with in situ hybridization data in human spinal cord tissue where *JUN* mRNA was found to be most abundant in MNs compared with other layers of the spinal cord, with further increases in transcript levels observed in spinal tissue of ALS patients (Virgo and de Belleroche, 1995). Hence, hyperactivation of JUN in ALS MNs may disrupt the homeostatic program, thereby driving degeneration. Since the protein levels of JUN in non-MNs are low, increased levels of JUN in non-MNs may not be sufficient to prove lethal.

In summary, by combining genome correction of SOD1 mutation in patient-derived iPSCs with deep RNA-seq, we have identified signaling pathways perturbed in ALS. Further, by pharmacologically inhibiting the upregulated pathways, we find that activation of the AP1 pathway, possibly via MAPK signaling, results in neurodegeneration of MNs. Our results exemplify the power of combining genomics and genome editing with iPSC-based disease models to elucidate mechanisms of neurodegeneration and also provide phenotypic screens to search for novel molecular targets.

## Experimental Procedures

### Culture and Genome Editing of iPSCs

ALS patient-derived iPSCs bearing *SOD1 E100G/+* (ND35662) mutation as well as iPSCs derived from a healthy individual (GM23280A) were obtained from the Coriell Institute for Medical Research. Cells were maintained as colonies on human ES qualified Matrigel (Corning) in mTeSR (STEMCELL Technologies). Plasmids for the hCas9 and gRNA were obtained from Addgene and were a kind gift from the Church lab (Mali et al., 2013). To enable efficient genome editing, we made an all-in-on vector that expressed the hCas9 from a CMV promoter and the guide-RNA targeting respective mutated loci from an U6 promoter. Mutant iPSCs were nucleofected with this vector along with a single-stranded DNA oligonucleotide homologous to 120 nt around the mutated site but bearing the wild-type sequence.

### Differentiation of iPSCs into Spinal Motor Neurons

We adapted a recently published protocol to differentiate iPSCs into spinal MNs (Maury et al., 2015). iPSCs were plated as colonies onto Matrigel and differentiated by treatment with neuronal differentiation media (N2B27: DMEM/F12, Neurobasal, N2 supplement 1%, B27 supplement 1%, L-glutamine 1%, ascorbic acid 5 μM, insulin 20 μg/mL) supplemented with SB431542 (80 μM), CHIR9921 (3 μM), and LDN8312 (0.2 μM) from day 0 until day 4. Cells were caudalized by treatment with 0.1 μM retinoic acid starting at day 2 and ventralized with 1 μM purmorphamine starting at day 4 and continued until day 10. At day 10, OLIG2-positive MN progenitors were re-plated onto poly-D-lysine/laminin-coated wells and differentiated by treating the cells with N2B27 media supplemented with brain-derived neurotrophic factor 20 ng/mL, GDNF 10 ng/mL, and DAPT 10 μM. DAPT treatment was stopped at day 15, and neuronal cultures were pulsed with mitomycin at a dose of 10 μg/mL for 1 hr to prevent further proliferation of any undifferentiated progenitors. Neuronal cultures were maintained until day 30 by changing the media every other day.

### Electrophysiology

For multi-electrode array (MEA) recordings, progenitors derived from GM23280A iPSCs were plated directly on an MEA and differentiated as described above. Extracellular electrophysiological recordings of D34 neurons were performed in neuronal growth medium using a 60-channel MEA platform (MEA1060, Multichannel Systems) as previously described (Dranias et al., 2013). MC_Rack software (Multichannel Systems) was used to acquire extracellular signals that were high-pass filtered at 300 Hz and low-pass filtered at 3 kHz with second-order Butterworth filters. Signals were sampled at 25 kHz. Action potentials or spikes were detected using voltage thresholds that were set at 6 SDs away from the channel's baseline noise. Electrophysiological data were imported into MATLAB using the Neuroshare API library (www.neuroshare.org). Optical stimulation was performed by the system setup as previously described (Ju et al., 2015). A 500 mW 473 nm blue laser beam was expanded and projected onto a reflective spatial light modulator (SLM, Holoeye Photonics), with a resolution of 1920 × 1080 pixels. The SLM received input from the computer to produce reflective patterns. The generated light patterns were then projected onto the MEA culture through the objective lens of an inverted microscope (Nikon Ti-E). The final light intensity at the MEA culture is ∼4.5 mW/mm^2^. Transistor-transistor logic pulses were sent to control the beginning and end of laser stimulus precisely.

### Calcium Imaging

GM23280A iPSC-derived D14 MNs were plated on glass bottom imaging dishes and infected with lentiviral particles expressing GCaMP6 for 6 hr, washed, and the media replaced with neuronal media as described above. At D21 of differentiation, cortical neurons obtained from embryonic day 18 (E18) rat pups were plated onto the MN monolayer. Cortical tissue was dissected from rat pups as described previously (Dranias et al., 2013). Cortical MN co-cultures were allowed to develop for 2 weeks before recording the GCaMP6 fluorescence intensity. After GCaMP6 recordings, co-cultures were treated with the Ca^2+^ indicator Fluo-4, incubated for 15 min, and fluorescence intensities were recorded for the same field.

### MN Survival Assay

Day 10 MN progenitors were plated on poly-D-lysine/laminin-coated 96-well plates at a density of 5,000 cells per well and differentiated into neurons. Cells were immunostained at day 30 (Supplemental Experimental Procedures) and images were captured on the Operetta imaging system (PerkinElmer) in an automated fashion. MNs and non-MNs were detected based on ISL1 and TUJ1 staining and quantified by the Columbus software using standard pipelines. Cells were cultured further for 14 days, immunostained, and counted as above. Counts at day 44 were normalized to those obtained at day 30. Motor neuronal soma and neurites were detected by first identifying ISL1+ nuclei and then demarcating soma and neuronal processes using Columbus pipelines. Apoptotic cells at day 37 were detected using the CellEvent Caspase 3/7 green detection reagent (Thermo Fisher) according to the manufacturer's instructions. The reagent was added to live neurons and incubated for 30 min, then counterstained with the nuclear dye Hoechst 33542 (Molecular Probes).

### RNA-Seq and Analysis

RNA was extracted from day 30 neuronal cultures as described above. Sequencing libraries were generated from 1 μg of RNA using the NEBnext Ultra kit (NEB) according to the manufacturer's instructions and sequenced on an Illumina HiSeq instrument. Raw reads were mapped to the human genome hg19 using bwa, and only reads mapping to unique locations retained. The DESeq2 R package (Love et al., 2014) and custom R scripts were used to analyze the mapped reads and generate a list of differentially expressed genes sorted by the statistical score. GSEA was performed using the GSEA software (http://software.broadinstitute.org/gsea/index).

### Immunofluorescence

For immunostaining, cells were fixed with 4% paraformaldehyde, permeabilized with ice-cold methanol for 5 min, and washed with PBS containing 10% serum for 1 hr at room temperature. Cells were incubated with primary antibodies (Table S2) diluted into PBS containing 10% serum and incubated overnight at 4°C. Next day, the cells were washed and incubated with Alexa-Fluor conjugated secondary antibodies (Molecular Probes) for 45 min at room temperature, and nuclei were stained with Hoechst 33542 (Molecular Probes).

### Western Blot

Cell lysates were separated on 12% SDS-PAGE gels, and proteins were transferred onto polyvinylidene fluoride (PVDF) membranes. Membranes were blocked with 5% milk in TBST (25 mM Tris [pH 8.0], 150 mM NaCl, 0.05% Tween 20) and probed with corresponding primary antibodies against specific proteins (Table S2). Horseradish-peroxidase-conjugated secondary antibodies (SCBT) were used to detect primary antibodies, and proteins were visualized by chemiluminescence (Nacalai Tesque). The SOD1 soluble fraction (TBS + 1% Triton X-100) and insoluble fraction (TBS + 1% Triton X-100 + 5% SDS) were transferred to PVDF membranes and probed with an anti-SOD1 antibody.

### qRT-PCR

RNA was extracted with TRIzol reagent (Invitrogen) and reverse transcribed using random hexamers and the high-capacity reverse transcription system from Applied Biosystems. PCR was performed using the SYBR GREEN PCR Master Mix from Applied Biosystems. The target gene mRNA expression was normalized to the expression of GAPDH, and relative mRNA fold changes were calculated by the ΔΔCt method. Primer sequences have been included in Table S3.

## Author Contributions

Conception and Design, A.B., L.W.S.; Collection and Assembly of Data, A.B., S.C.N.; Data Analysis and Interpretation, A.B., S.C.N., L.W.S.; Manuscript writing, A.B., S.C.N., L.W.S. Financial Support, L.W.S. X.Z. performed the MEA recordings and Ca^2+^ imaging and analyzed the data. A.M.J.V. analyzed and interpreted the MEA recordings and Ca^2+^ imaging data and contributed to the experimental design.

## Accession Numbers

RNA-seq data are available at www.ncbi.nlm.nih.gov/sra: SRA: SRX2494130, SRX2494129, SRX2494128, SRX2494127.

## Supplemental Information

Document S1. Supplemental Experimental Procedures, Figures S1–S3, and Tables S1–S3

Document S2. Article plus Supplemental Information
