# Supplementary material for: Genetic Correction of SOD1 Mutant iPSCs Reveals ERK and JNK Activated AP1 as a Driver of Neurodegeneration in Amyotrophic Lateral Sclerosis
Source: Stem Cell Reports. 2017 Mar 30;8(4):856–69. doi: 10.1016/j.stemcr.2017.02.019 (PMC5390134; doi:10.1016/j.stemcr.2017.02.019)
Supplement: Document S1. Supplemental Experimental Procedures, Figures S1–S3, and Tables S1–S3 [file mmc1.pdf]

Stem Cell Reports, Volume *8*

Supplemental Information

Genetic Correction of SOD1 Mutant iPSCs Reveals ERK and JNK Activated AP1 as a Driver of Neurodegeneration in Amyotrophic Lateral Sclerosis

Akshay Bhinge, Seema C. Namboori, Xiaoyu Zhang, Antonius M.J. VanDongen, and Lawrence W. Stanton

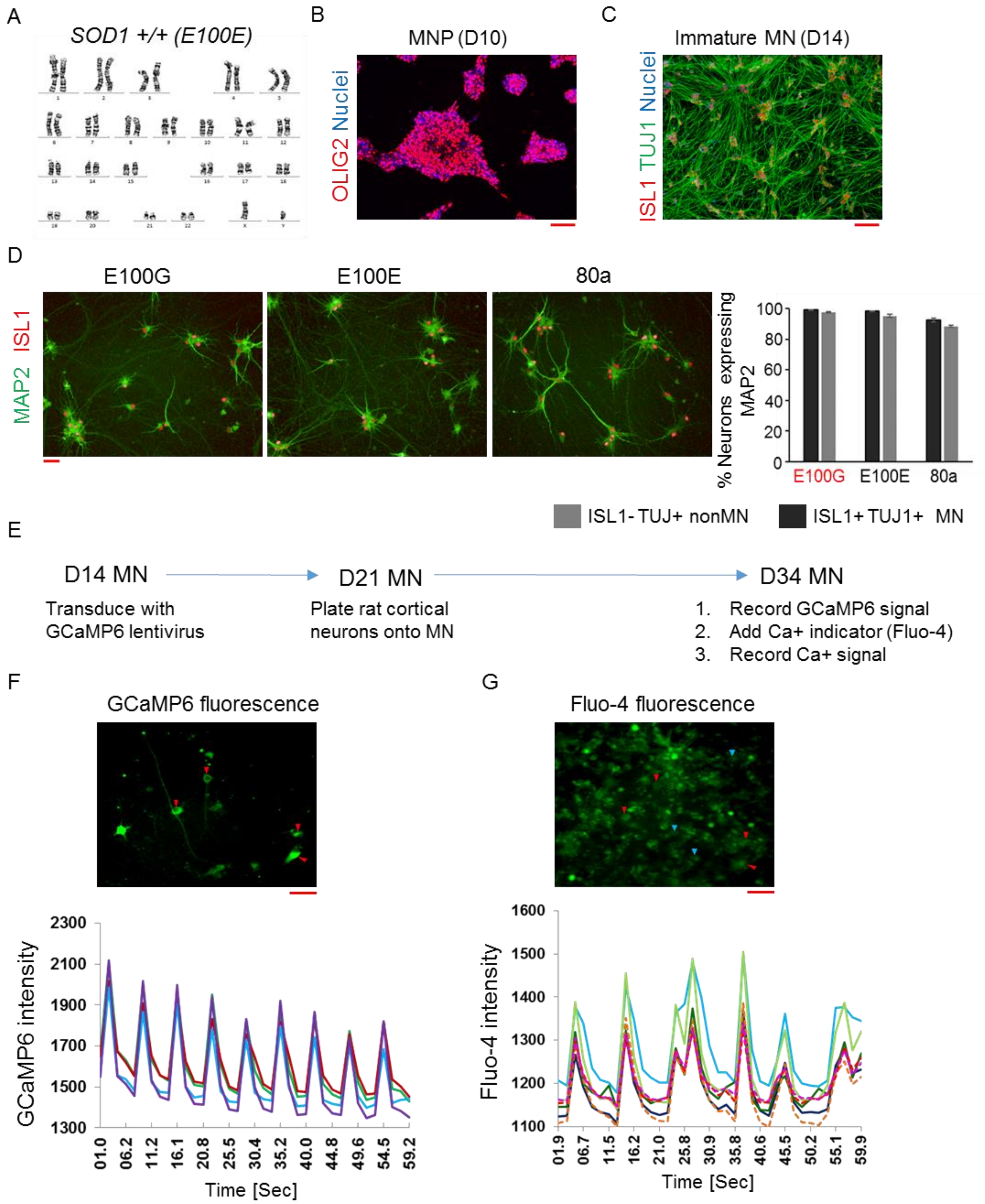

Figure S1. Genome editing and differentiation of iPSC into spinal MN (related to Figure 1)

(A) Karyogram for *SOD1 +/+* genome corrected iPSC displaying a normal karyotype. (B) Representative image of OLIG2+ motor neuron progenitors generated at d10. Scale bar indicates 100µM. (C) Representative image of MN at d14 stained for ISL1 (red), TUJ1 (green) and Nuclei (blue). Scale bar indicates 100µM. (D) Quantitation

of ISL1+ and ISL1- neurons expressing MAP2 at d30. (n=3, error bars indicate SEM). Scale bar indicates 50µM. (E) Experimental schema for the cortical-motor neuron co-culture recordings. (F) GCaMP6 fluorescence intensities plotted as a function of time. Representative traces have been displayed for 4 MN marked with red arrows. Scale bar indicates 50µM. (G) Same field visualized after applying Ca^{2+} indicator Flou-4. Signal for cortical neurons (blue arrows) has been plotted as dashed lines. Signal for motor neurons (red arrows) has been plotted as solid lines. Scale bar indicates 50µM.

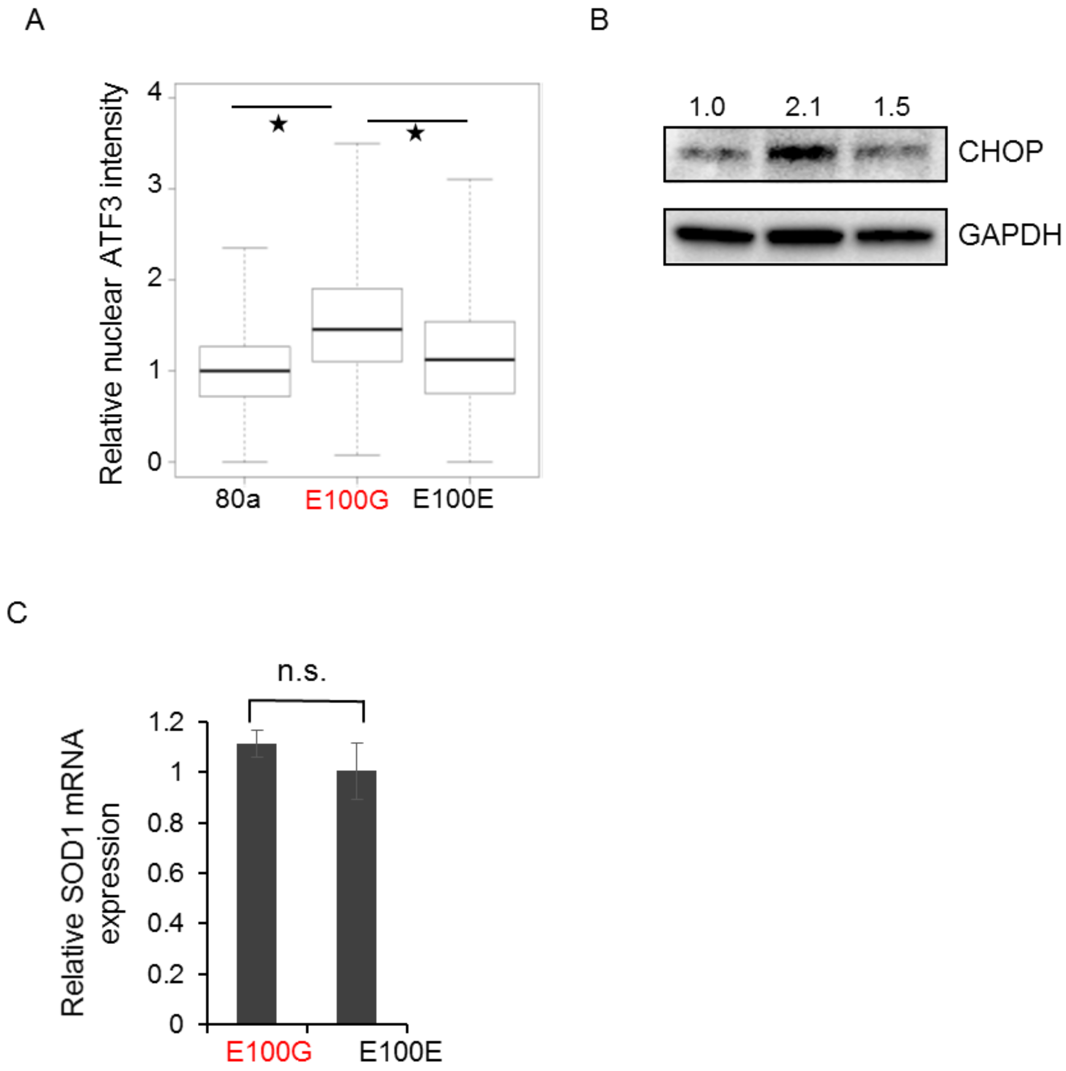

Figure S2. Mutant SOD1 MN display elevated levels of ER stress (related to Figure 2)

(A) Immunostaining reveals increased levels of nuclear ATF3 in SOD1 mutant MN compared to control and isogenic corrected MN. (B) Western blot assays display increased protein levels of CHOP in mutant MN compared to controls. (C) SOD1 transcript levels are unaltered between mutant SOD1 and isogenic corrected MN by quantitative RT-PCR (n=3, error bars indicate SEM, n.s. indicates not significant).

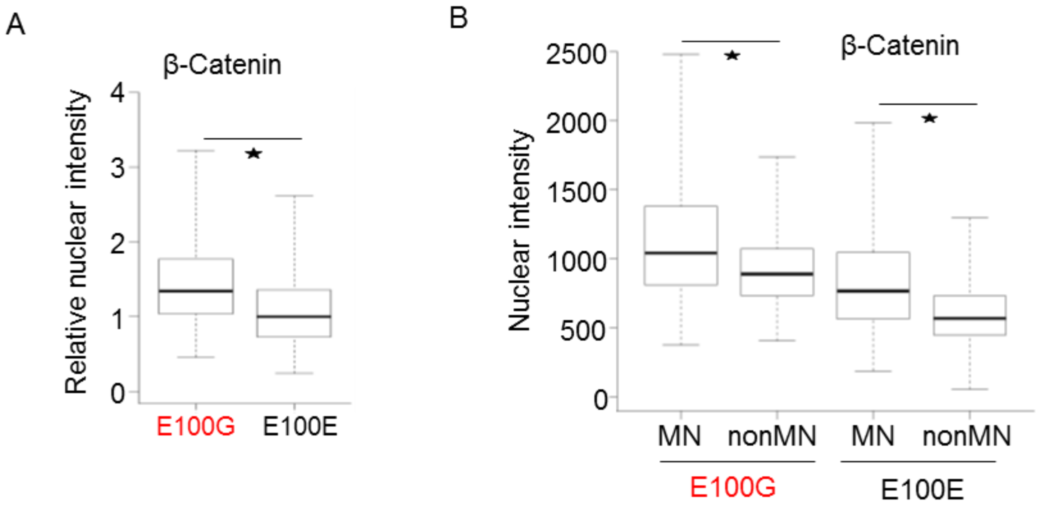

Figure S3. Activation of WNT in ALS MN (Related to Figure 4)

(A) Boxplot displaying nuclear intensities of β-Catenin in ALS mutant MN normalized to the respective isogenic controls. (B) Boxplot displaying nuclear intensities of β-Catenin in mutant SOD1 MN and non-MN as well as isogenic control MN and non-MN. Data from 3 independent replicates were pooled to generate the boxplot and estimate the p-values. ($p < 0.01$, two-tailed students t-test was used to estimate p-values)

Table S1. Small molecule inhibitors. (Related to Figure 4)

Chemical	Source	Pathway / Target
Pifithrin-α hydrobromide	Tocris	TP53
SB203580	Tocris	p38/MAPK
FR180204	SCBT	ERK 1,2
SP600125	SCBT	JNK 1,2,3
XAV 939	Tocris	WNT
AT7519	Selleckchem	CDK1,2,3,4,6,9

Table S2. Antibodies used in this study. (Related to Figures 1,2,4,5)

Target	Source	Catalog number
ISL1	Abcam	ab8650
ISL1	Abcam	ab109517
TUJ1	Abcam	ab107216
MAP2	Abcam	ab11267
CHAT	Millipore	ab144P
p-JUN	Cell Signaling Technology	9261
β-CATENIN	Abcam	ab16051
TP53	SCBT	sc-126
p-ERK	SCBT	sc-7383
ERK	SCBT	sc-94
p-p38	Cell Signaling Technology	9211
p38	Cell Signaling Technology	8690
p-JNK	Cell Signaling Technology	9251
JNK	Cell Signaling Technology	9252
SOD1	SCBT	sc-17767
GAPDH	Millipore	MAB374

Table S3. Primer sequences. (Related to Figure 2)

Target	Sequence	Reference
**SOD1**	F: AGTGCAGGTCCTCACTTTA<br>R: CCTGTCTTTGTACTTTCTTC	**(Towne et al., 2008)**
**XBP1**	F: GGAGTTAAGACAGCGCTTGG<br>R: CACTGGCCTCACTTCATTCC	**(Ng et al., 2015)**
**sXBP1**	F: TGCTGAGTCCGCAGCAGGTG<br>R: GCTGGCAGGCTCTGGGGAAG	**(Ng et al., 2015)**

Supplemental Experimental Procedures

Genome editing patient-derived iPSC

About 8x 10e5 patient derived iPSC were nucleofected with 4ug of plasmid and 4ug of a 121 nucleotide single stranded DNA oligo that served as the donor for the reference allele. Nucleofection was performed on an Amaxa nucleofector IIb with the protocol for H1 ES cells according to the manufacturer's instructions. Since the plasmid expressed a GFP, nucleofected cells that were GFP+ cells were sorted by flow cytometry, 40hrs post-transfection and collected into mTeSR supplemented with Rock inhibitor (10µM) and pen-strep antibiotics. Cells were plated into 6-well plates at density of 750 cells/cm2 and cultured for 10 days till well-separated colonies were observed. Individual colonies were picked and plated into 96-well plated for further culture and screening.

For both the SOD1 and FUS iPSC, ~180 colonies were screened. We identified 1 "clean" homozygous correction and 2 mixed clones for the SOD1 iPSC. The mixed clones were frozen and not analysed further. The homozygous clone was further passaged and the genotype confirmed for at least 7 passages. We did not genotype future passages. We obtained 1 homozygous and 1 heterozygous correction for the FUS iPSC. However, the homozygous clone had a single nucleotide insertion that caused a frameshift and had to be discarded. The heterozygous clone was genotyped for 6 passages and was found to be stable.

Supplemental references

Ng SY, Soh BS, Rodriguez-Muela N, Hendrickson DG, Price F, Rinn JL, Rubin LL (2015) Genome-wide RNA-Seq of Human Motor Neurons Implicates Selective ER Stress Activation in Spinal Muscular Atrophy. *Cell stem cell* **17:** 569-584

Towne C, Raoul C, Schneider BL, Aebischer P (2008) Systemic AAV6 delivery mediating RNA interference against SOD1: neuromuscular transduction does not alter disease progression in fALS mice. *Molecular therapy : the journal of the American Society of Gene Therapy* **16:** 1018-1025
